# A hypoxia-on-a-chip platform for modeling ischemic arrhythmogenesis and evaluating the effects of levosimendan and OR-1896 on ischemic human iPSC-derived cardiomyocytes

**DOI:** 10.3389/fbioe.2025.1671013

**Published:** 2025-09-29

**Authors:** Mahmoud Gaballah, Kaisla Walls, Fatma Zakzook, Joose Kreutzer, Jouko Levijoki, Katriina Aalto-Setälä

**Affiliations:** ^1^ Heart Group, Faculty of Medicine and Health Technology, Tampere University, Tampere, Finland; ^2^ Department of Forensic Medicine and Toxicology, Faculty of Veterinary Medicine, University of Sadat City, Menoufia, Egypt; ^3^ Computational Biophysics and Imaging Group, Faculty of Medicine and Health Technology, Tampere University, Tampere, Finland; ^4^ Department of Forensic Medicine and Toxicology, Faculty of Veterinary Medicine, Kafrelsheikh University, Kafrelsheikh, Egypt; ^5^ BioGenium Microsystems Ltd, Tampere, Finland; ^6^ Orion Corporation Orion Pharma, Espoo, Finland; ^7^ Heart Hospital, Tampere University Hospital, Tampere, Finland

**Keywords:** calcium cycling by antiarrhythmic effect, hypoxia by Ischemia modeling, human-induced pluripotent stem cell-derived cardiomyocytes, calcium cycling, hypoxia, levosimendan, levosimendan metabolite OR-1896, cardioprotection

## Abstract

Acute hypoxia is a major contributor to cardiomyocyte damage and dysfunction in ischemic heart disease, and the effective therapeutic strategies remain limited. Levosimendan, a calcium sensitizer with both inotropic and vasodilatory effects, along with its active metabolite OR-1896, is utilized in the treatment of acute heart failure. In this study, we investigated the cardioprotective and antiarrhythmic effects of levosimendan and its metabolite OR-1896 under hypoxic conditions using human-induced pluripotent stem cell-derived cardiomyocytes (hiPSC-CMs). hiPSC-CMs were exposed to acute hypoxia and treated with levosimendan or its metabolite OR-1896. Structural integrity was assessed via immunostaining and electron microscopy imaging. Calcium transient abnormalities were evaluated using live-cell imaging. Hypoxia-induced injury was further assessed by measuring cardiac biomarkers and gene expression profiling of hypoxia-associated pathways. Hypoxia induced significant structural damage, including sarcomere disorganization, mitochondrial cristae fragmentation, and nuclear shrinkage, accompanied by increased release of cardiac biomarkers. Hypoxia also upregulated genes associated with the hypoxia response, oxidative stress, and apoptosis, while disrupting calcium handling and increasing arrhythmic events. Treatment with levosimendan and its metabolite OR-1896 preserved cellular structure, reduced biomarker release, and stabilized calcium transients, significantly reducing hypoxia-induced arrhythmogenesis. Both compounds also modulated gene expression, downregulating hypoxia-responsive and oxidative stress markers, and inhibiting apoptotic pathways. Notably, the metabolite OR-1896 exhibited protective effects comparable to or even greater than those of levosimendan. This study provides the first comprehensive evidence of the cardioprotective and antiarrhythmic properties of levosimendan’s metabolite, demonstrating its ability to reduce hypoxia-induced cellular injury and correct abnormal Ca^2+^ transients. These findings highlight the therapeutic potential of levosimendan and its clinically significant long-acting metabolite, OR-1896, in the treatment of cardiac ischemia.

## 1 Introduction

Ischemic heart disease (IHD) remains the leading cause of morbidity and mortality worldwide (Tsao et al., 2022). It occurs when coronary arteries narrow due to plaque accumulation, reducing blood flow to the myocardium and potentially forming ischemic regions within the heart tissue. The most severe manifestation of IHD is acute myocardial infarction (AMI), which typically results from the sudden rupture of a plaque. This rupture triggers thrombus formation, potentially causing complete occlusion of the coronary artery. AMI induces irreversible myocardial damage within hours of onset and can result in life-threatening complications, including sudden cardiac death, and heart failure (Buja, 2023), (Reed et al., 2017).

The development of experimental models to study human AMI is essential for advancing new therapeutic strategies. Currently, the primary treatment for AMI involves restoring blood flow to the occluded coronary artery through percutaneous coronary intervention or fibrinolytic therapy (O’Gara et al., 2013). However, AMI also causes cellular damage, creating a favorable environment for life-threatening arrhythmias such as ventricular tachycardia and fibrillation (Frampt et al., 2023), underscoring the need for robust models to investigate these mechanisms. Currently, arrhythmias during AMI are primarily prevented with β-blockers (Joo, 2023), while severe cases are treated with defibrillation (O’Gara et al., 2013). Other available antiarrhythmic drugs, apart from β-blockers, are not recommended for prophylactic use and have shown severe side effects and limited efficacy when used to treat arrhythmias during AMI (Piccini et al., 2011). Consequently, there is an urgent need to develop novel, potent therapeutics to prevent cellular damage and mitigate arrhythmic events associated with AMI effectively.

Levosimendan is a calcium sensitizer with positive inotropic properties, currently utilized in clinical settings for the management of acute decompensated heart failure (Haik et al., 1992), (Nandkeolyar et al., 2021). It exerts its effects by stabilizing the Ca^2+^-bound form of cardiac troponin C (cTnC), which enhances the sensitivity of cardiac myofilaments to Ca^2+^ during systole, thereby improving myocardial contraction with minimal impact on energy consumption (Haikala et al., 1995). In acute heart failure, its benefits are primarily mediated through the activation of ATP-sensitive potassium (K_ATP) channels. By opening K_ATP channels in vascular smooth muscle, Levosimendan induces vasodilation, reducing afterload and improving blood flow, which ultimately decreases the workload on the heart (Pathak et al., 2013). Following administration, levosimendan is metabolized within an hour by intestinal bacteria into the intermediate metabolite OR-1855, which is subsequently acetylated to form the biologically active metabolite OR-1896 (Antila et al., 1999), (Kivikko et al., 2002).

Animal models have traditionally been employed to study cardiac phenotypes in both health and disease. However, significant differences in physiology and electrochemical properties between animals and humans limit their translational relevance (Vuorenpää et al., 2023). The advent of hiPSC-CMs has provided a continuous and reliable source of human-based cardiomyocytes (CMs), enabling more accurate modeling of cardiac diseases. hiPSC-CMs not only recapitulate key physiological features of the human heart but also retain the genetic background of the donor and exhibit drug sensitivity corresponding to human responses (Penttinen et al., 2015), (Wei et al., 2019). Although challenges related to cell maturity remain, hiPSC-CMs have been successfully utilized in various experimental setups to model myocardial ischemia (Häkli et al., 2021), (Hidalgo et al., 2018), (Häkli et al., 2022).

In this study, we aimed to investigate the antiarrhythmic and cellular protective effects of Levosimendan’s main metabolite, OR-1896, using a hiPSC-CM model of ischemia, building on our previous findings (Gaballah et al., 2022). We assessed cell morphology, calcium handling properties, protein expression, and gene expression in hypoxia-exposed hiPSC-CMs, comparing them to hypoxia-exposed hiPSC-CMs treated with levosimendan and its metabolite OR-1896. Both levosimendan and its metabolite OR-1896 demonstrated significant cardioprotective effects, as evidenced by preserved cell morphology resembling normoxic conditions and reduced release of cardiac markers for cellular damage. Furthermore, levosimendan and its metabolite OR-1896 exhibited potent antiarrhythmic properties, characterized by a decreased incidence of abnormal Ca^2+^ transients. Gene expression analysis provided additional insights into the molecular mechanisms underlying these protective effects. These findings highlight the potential of levosimendan and its metabolite OR-1896 as therapeutic agents for mitigating ischemia-induced arrhythmias and cellular injury.

## 2 Materials and methods

### 2.1 Generation and characterization of human induced pluripotent stem cells

This study was conducted in accordance with the guidelines of the Ethics Committee of Pirkanmaa Hospital District (Aalto-Setälä R08070) for the establishment, culturing, and differentiation of human induced pluripotent stem cell (hiPSC) lines. The previously established UTA.04602.WT cell line, derived from a healthy 55-year-old female, was utilized in this study (Ojala et al., 2012). The undifferentiated hiPSC line was generated through retroviral reprogramming of human fibroblasts using the transcription factors OCT4, SOX2, KLF4, and c-MYC. The resulting hiPSCs were validated for pluripotency and karyotypic normality (Ohnu et al., 2009). For culturing, hiPSCs were maintained on mouse embryonic fibroblast (MEF) feeder cells (Gibco) in KnockOut Serum Replacement (KSR) medium. The KSR medium consisted of KnockOut DMEM (Gibco) supplemented with 10% KnockOut Serum Replacement (Gibco), 1% MEM Non-Essential Amino Acids (NEAA, Gibco), 1% GlutaMAX (Gibco), 0.2% β-mercaptoethanol (Gibco), and 0.5% penicillin/streptomycin (Lonza).

### 2.2 hiPSCs differentiation and hiPSC-CMs sorting

**FIGURE 1:**
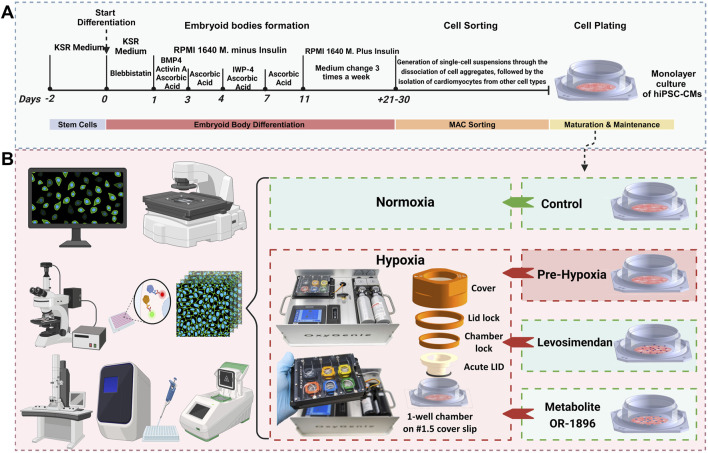
Differentiation protocol and experimental workflow. **(A)** A schematic overview diagram illustrates the methodology for differentiating hiPSCs into CMs using the EB method. **(B)** A schematic overview diagram illustrates experimental workflow: The normoxic control group was maintained under normoxic conditions throughout the experiment. For Ca^2+^ imaging, hypoxia was induced for 7 h in the hypoxia and treatment groups, with images captured at 0, 4, and 7 h. For immunostaining, electron microscopy, and gene expression studies, hypoxia was induced for 24 h. Media were collected for ELISA analysis prior to cell fixation. Gene expression samples were collected after 24 h of hypoxia.

Cardiomyocyte differentiation was performed using the embryoid body (EB) method, as previously described (Prajapati et al., 2021), with minor modifications (see Supplementary Information for the full protocol) (Figure 1A). After 21 days, magnetic-activated cell sorting (MACS) was employed to remove non-cardiomyocyte cells prior to plating. Single cells were obtained using the MultiTissue Dissociation Kit 3 (Miltenyi Biotec) according to the manufacturer’s instructions. CMs were subsequently isolated from other cell types using the PSC-Derived Cardiomyocyte Isolation Kit, human (Miltenyi Biotec), following the manufacturer’s protocol, as described previously (Pekkanen-et al., 2019). The isolated CMs were resuspended in 20% EB medium, which consisted of KnockOut DMEM supplemented with 20% fetal bovine serum (FBS, Gibco), 1% MEM Non-Essential Amino Acids (NEAA), 1% GlutaMAX, and 0.5% penicillin/streptomycin. The cells were then plated onto the glass base (#1.5 cover slip glass) of an OxyGenie 1-well culture chamber (BioGenium Microsystems Ltd, Tampere, Finland) pre-coated with 0.1% gelatin. A cell density of 100,000–150,000 cells per chamber was used to achieve a monolayer. Cultures were maintained in a humidified incubator at 37 °C and 5% CO_2_. The cells were cultured in the chambers for approximately 7–10 days before hypoxia experiments were conducted. During this period, the culture medium was replaced every other day.

### 2.3 Experimental setup

Each experiment consisted of four groups: (1) normoxic control, (2) hypoxic control without treatment, (3) hypoxia with levosimendan, and (4) hypoxia with the levosimendan metabolite OR-1896 (Figure 1B). Serum-free EB medium was used for all groups, and media changes were performed uniformly across conditions. Levosimendan (Sigma-Aldrich) and its active metabolite OR-1896 (Orion Corporation, Finland) were solubilized in dimethyl sulfoxide (DMSO; Life Technologies) to a stock concentration of 10 mM. These stock solutions were further diluted in serum-free EB medium (KnockOut DMEM supplemented with 1% MEM Non-Essential Amino Acids (NEAA), 1% GlutaMAX, and 0.5% penicillin/streptomycin) to achieve the final working concentrations immediately before application to the cells. A concentration of 2 μM was selected for both levosimendan and its metabolite OR-1896. The final concentrations of levosimendan and its metabolite OR-1896 were prepared in serum-free EB medium just prior to the final media change and drug administration. The drugs were allowed to incubate for 15 min before initiating the hypoxia experiments. The normoxic control group was maintained under normoxic conditions for the entire duration of the experiment. For Ca^2+^ imaging, hypoxia was induced for a total of 7 h in the hypoxia group and the other treatment groups. Images were captured at baseline (0 h), 4 h, and 7 h for all four groups to compare the changes in calcium cycling of the CMs. After hypoxia exposure, samples were carefully processed to avoid additional reoxygenation injury. For immunostaining imaging and electron microscopy imaging, hypoxia was induced for 24 h in the hypoxia group and the other treatment groups. Following hypoxia exposure, media were collected from samples for ELISA analysis prior to cell fixation. The cells were then fixed for imaging to evaluate structural changes in the CMs. For gene expression studies, samples were collected after 24 h of hypoxia in the hypoxia group and the other treatment groups to assess changes in gene expression levels. To determine the appropriate experimental window for hypoxia modeling, we initially examined CMs subjected to 30 h of hypoxia. Immunocytochemistry revealed extensive cell death at this timepoint, including severe sarcomeric disorganization, and cytoskeletal disruption (Supplementary Figure 1). Due to this excessive damage, electrophysiological and contractility analyses were not feasible. Therefore, we selected 24 h of hypoxia exposure as the optimal experimental duration, as it allowed us to capture functional changes while minimizing cell loss.

### 2.4 Hypoxia setup

The OxyGenie mini-incubator platform (BioGenium Microsystems Ltd, Tampere, Finland) was used to induce hypoxia, as previously described (Gaballah et al., 2022). In brief, the portable OxyGenie mini-incubator facilitates extended gas- and temperature-controlled experiments outside conventional incubators. The system includes a temperature controller, refillable gas inlets, an indium tin oxide (ITO) transparent heater plate, a Flow Divider for six culture chambers, and one to six individual 1-well culture chambers made of silicone elastomer attached to a #1.5 coverslip glass, along with corresponding covers and lids. Unlike previous experiments, a specialized Acute LID was used instead of a solid glass lid. This hollow lid incorporates a gas-permeable membrane, enabling rapid oxygen depletion within minutes to mimic acute hypoxia. The Acute LID assembly was slightly modified from the previous study (Häkli et al., 2022) to improve the efficiency of rapid oxygen exchange and enhance repeatability. In this study, hypoxia was induced using a premixed gas containing 0% O_2_, 5% CO_2_, and 95% N_2_, supplied to the device at a flow rate of 5 mL/min/1-well chamber. Upon completion of the hypoxia experiment, the chambers were individually detached from the OxyGenie mini-incubator for further analysis.

### 2.5 Immunocytochemistry

For immunocytochemistry, hypoxic samples for the hypoxia and treatment groups were maintained under hypoxia for 24 h, while normoxic control samples were kept under normoxic conditions for the same duration. After the incubation period, all samples were fixed with 4% paraformaldehyde (PFA; Sigma-Aldrich) for 20 min. The samples were then blocked with a blocking solution (10% normal donkey serum (NDS), 0.1% Triton X-100, and 1% bovine serum albumin (BSA) in phosphate-buffered saline (PBS)) for 45 min at room temperature (RT). Primary antibodies were used to stain the samples as follows: hypoxia-inducible factor 1 alpha (HIF-1α) with a recombinant HIF1A monoclonal antibody (rabbit IgG, 1:200; ThermoFisher), α-actinin with a monoclonal anti-α-actinin antibody (mouse IgG, 1:500; Sigma-Aldrich), troponin T with monoclonal cardiac troponin T antibody (rabbit IgG, 1:400; Abcam), and myosin-binding protein C (mouse IgG, 1:200; Santa Cruz Biotechnology). The primary antibodies were incubated overnight at +4 °C. After incubation, the samples were washed three times with 1% BSA in PBS. Secondary antibodies, donkey anti-mouse Alexa Fluor™ 568 (1:1000; Invitrogen) and donkey anti-rabbit Alexa Fluor™ 488 (1:1000; Invitrogen), were added and incubated for 1 h at room temperature in the dark. Finally, cell nuclei were stained using VECTASHIELD mounting medium with DAPI (Vector Laboratories). Cells were observed under an Olympus IX51 fluorescence microscope, and fluorescent image analysis was performed using ImageJ software (National Institutes of Health, Bethesda, MD, United States).

### 2.6 Electron microscopy

For normoxic conditions, cultured hiPSC-CMs were washed with phosphate-buffered saline (PBS) and fixed in a solution containing 2.5% glutaraldehyde and 2% paraformaldehyde in 0.1 M PBS for 30 min. For hypoxic samples, hiPSC-CMs were fixed after 24 h of hypoxia using the same fixation protocol. After fixation, the buffer was washed away using 0.1 M sodium phosphate buffer. The samples could then be stored in 2% paraformaldehyde in 0.1 M sodium phosphate buffer until further processing, ensuring that they remained hydrated to preserve cellular structures. hiPSC-CMs were post-fixed with 1% osmium tetroxide for 1 h. After post-fixation, the cells were dehydrated through a graded ethanol series. The coverslip was then dipped in acetone and placed onto an aluminum planchette. Epon was immediately added to cover the cells, providing the stability necessary for ultrathin sectioning. A beem capsule filled with Epon was placed upside down over the cells and incubated for 2 h to allow the Epson to infiltrate the cells. After incubation, the samples were baked at 60 °C overnight. The following day, samples were transferred directly from the oven to a hot plate, where the coverslip was carefully removed, ensuring that no glass fragments remained attached. Ultrathin sections (approximately 60 nm thick) were cut using an ultramicrotome with diamond knives (Leica EM Ultracut UC6i or UC7, Leica Mikrosysteme GmbH, Austria) and collected onto copper grids. The sections were stained with uranyl acetate and lead citrate to enhance contrast, particularly for organelle membranes and other ultrastructural details. Finally, the samples were examined, and images were captured using a JEM-1400PLUS transmission electron microscope (JEOL Ltd, Japan) equipped with an Orius SC 1000B bottom-mounted CCD camera (Gatan). Images were acquired at an accelerating voltage of 80 kV, with magnifications ranging from ×1,000 to 100,00×.

### 2.7 Ca^2+^ imaging

Ca^2+^ transients were studied using the fluorescent calcium indicator Cal-520^®^ AM (AAT Bioquest), which provides high sensitivity and signal-to-noise ratio for detecting intracellular Ca^2+^ dynamics. Cells were incubated with 5 µM Cal-520^®^ AM in pre-warmed extracellular solution (ECS) for 90 min at 37 °C to ensure efficient dye loading and intracellular ester hydrolysis. The ECS was freshly prepared in MilliQ H_2_O and consisted of 137 mM NaCl, 5 mM KCl, 0.44 mM KH_2_PO_4_, 20 mM HEPES, 4.2 mM NaHCO_3_, 5 mM D-glucose, 2 mM CaCl_2_, 1.2 mM MgCl_2_, and 1 mM Na-pyruvate. The pH was adjusted to 7.4 using NaOH to mimic physiological conditions. After dye loading, cells were gently washed twice with warm ECS to remove excess dye and reduce background fluorescence. Serum-free EB medium was then added to each imaging chamber, with or without pharmacological agents depending on the experimental group. To minimize medium evaporation, acute LIDs were placed on the OxyGenie one-well culture chambers. Cells were equilibrated for 15 min at 37 °C under normoxic conditions before baseline recordings (0 h). Normoxic control chambers were never exposed to hypoxic gas, while hypoxia and treatment groups were supplied with continuous hypoxic gas from a small tank through a single tubing line connected to the one-well chamber. Chambers were maintained at 37 °C throughout imaging. Fluorescence recordings were performed on an Axio Observer A1 inverted microscope equipped with a Fluar 20×/0.75 M27 objective (Carl Zeiss, Jena, Germany), an Andor iXon3 885 EM-CCD camera (Andor Technology, Belfast, United Kingdom), and a Lambda DG-4 Plus high-speed wavelength switcher (Sutter Instrument, Novato, CA, United States). The Zeiss filter set 69 was used with Cal-520^®^ AM excitation at 480 nm and emission captured at 540 nm. Image sequences of 30–60 s were acquired at 30 frames per second using Zen 2.3 (blue edition) software. For data analysis, regions of interest (ROIs) were manually selected from single spontaneously beating CMs or small clusters within the monolayer. Background fluorescence was measured from cell-free regions and subtracted before analysis. Fluorescence intensity (F) was normalized to the baseline fluorescence (F_0_), and Ca^2+^ transient parameters were expressed as ΔF/F_0_. Parameters quantified included peak amplitude, rise time (10%–90%), decay time (90%–10%), and beating frequency (beats per minute). Raw Ca^2+^ traces were exported and analyzed using Clampfit v10.5 (Molecular Devices, Silicon Valley, CA, United States). At least 20–30 cells per group from independent differentiations were analyzed to ensure biological reproducibility. All measurements were normalized to baseline recordings to allow direct comparison between groups (normoxia, hypoxia, and hypoxia with treatment).

### 2.8 Enzyme-linked immunosorbent assay

Enzyme-Linked Immunosorbent Assay (ELISA) was performed to measure cardiac troponin T (cTnT) using the Invitrogen™ Troponin T (TNNT1) Human ELISA Kit (EHTNNT1, Invitrogen) and N-terminal prohormone of brain natriuretic peptide (proBNP) using the Invitrogen™ Human proBNP/NPPB ELISA Kit (EHPRONPPB, Invitrogen). The assays were conducted in accordance with the manufacturer’s protocols. Notably, no dilution of the cell culture media was required, allowing for direct analysis of the samples. Each sample was measured in triplicate for both assays to ensure data accuracy and reproducibility. Protein absorbance was measured using a Tecan Infinite F200 Fluorescence Microplate Reader (Tecan) at a wavelength of 410 nm. Protein concentrations were determined using a Four-Parameter Logistic Curve generated by MyAssays.com.

### 2.9 Quantitative reverse transcription polymerase chain reaction (qRT-PCR) analysis

Quantitative reverse transcription polymerase chain reaction (qRT-PCR) was performed to evaluate gene expression in hypoxia-exposed hiPSC-CMs and those treated with levosimendan and its metabolite OR-1896, in comparison to normoxic controls. The research aimed to elucidate the effects of hypoxia on CM gene expression and explore the potential protective roles of levosimendan and its metabolite OR-1896 in mitigating hypoxic stress. Samples were collected in Monarch^®^ RNA Lysis Buffer (New England BioLabs, United States) and stored at −80 °C until further processing. Total RNA extraction was performed using the Monarch Total RNA Miniprep Kit (New England BioLabs, United States) according to the manufacturer’s instructions. Complementary DNA (cDNA) synthesis was carried out using a High-Capacity cDNA Reverse Transcription Kit (Applied Biosystems, United States) for each RNA sample. qPCR was conducted using the 96-well Standard TaqMan^®^ Array Human Hypoxia (Applied Biosystems, United States) in accordance with the manufacturer’s protocol, with TaqMan^®^ Gene Expression Master Mix (Thermo Fisher Scientific). The list of genes grouped according to their primary biological functions: Hypoxia response and HIF pathway, Metabolism and Oxidative stress, Apoptosis, and Transcriptional regulation (Supplementary Table 2). The reactions were performed on an ABI7300 thermal cycler (Applied Biosystems) under the following conditions: initial denaturation at 95 °C for 10 min, followed by 40 cycles of 95 °C for 15 s and 60 °C for 1 min. The cycle threshold (Ct) expression data was normalized to the housekeeping gene glyceraldehyde 3-phosphate dehydrogenase (GAPDH) and analyzed using the comparative ΔCt method. Relative gene expression changes were determined using the 2^−ΔΔCt^ method, comparing the hypoxia and treatment groups to the normoxic control, as previously described (Livak and Schmittgen, 2001).

### 2.10 Statistical analysis

Data are presented as mean ± standard errors of the mean (SEM). Statistical analysis was conducted with IBM SPSS Statistics 26 (SPSS, Chicago, IL, United States) and GraphPad Prism (version 10.3.1, GraphPad Software. For gene expression and ELISA data, one-way analysis of variance (ANOVA) was used to compare differences between groups. Post-hoc analysis was performed using Tukey’s test to evaluate significant differences between specific groups. For Ca^2+^ imaging data, comparisons between groups at the same time point were analyzed using the non-parametric Kruskal–Wallis test, followed by pairwise comparisons with Dunn’s *post hoc* test and Bonferroni corrections for multiple comparisons. Cases of arrhythmic regions of interest (ROIs) were tested for significance using the binomial Chi-squared test for all groups. Pairwise comparisons were further evaluated using Fisher’s exact test with Bonferroni corrections.

## 3 Results

### 3.1 Effect of hypoxia on the structure of hiPSC-CMs and the protective role of levosimendan and its metabolite OR-1896

**FIGURE 2:**
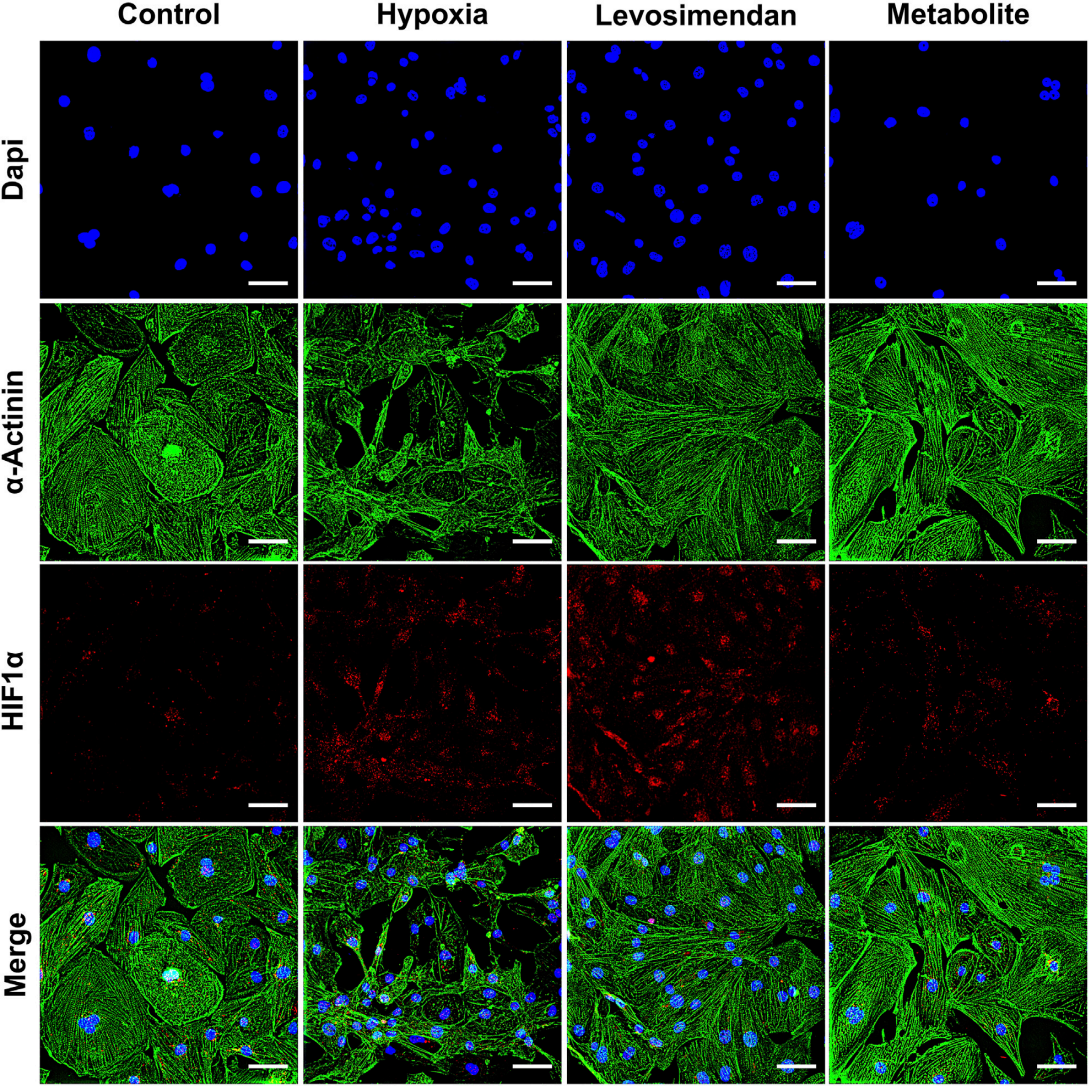
Immunostaining of α-actinin and HIF-1α in hiPSC-CMs. Representative images of α-actinin and HIF-1α staining in normoxic hiPSC-CMs, hypoxia-exposed hiPSC-CMs, and hypoxia-exposed hiPSC-CMs treated with levosimendan or its metabolite OR-1896. The scale bar represents 20 µm. *n* = 5 independent differentiations, with at least 25 random fields analyzed per replicate.

We evaluated the morphological and structural integrity of hypoxia-exposed hiPSC-CMs and hypoxia-exposed hiPSC-CMs treated with levosimendan and its metabolite OR-1896, comparing these groups to normoxic controls. Structural integrity was assessed using immunostaining for α-actinin (Figure 2), troponin T (Supplementary Figure 2), and myosin-binding protein C (Supplementary Figure 3). In normoxic condition, hiPSC-CMs exhibited intact structural features, including well-aligned sarcomeres and a consistent organization of the cytoskeletal framework. In contrast, hypoxia-exposed hiPSC-CMs showed significant structural disorganization, including misaligned sarcomeres, disrupted cytoskeletal patterns, and compromised structural integrity. These changes were accompanied by irregular cell morphology, such as cell shrinkage and detachment. Importantly, hypoxia-exposed hiPSC-CMs treated with levosimendan and its metabolite preserved sarcomere alignment and prevented structural degradation induced by hypoxia. Both treatments stabilized α-actinin, troponin, and myosin filaments, protecting the cellular framework and maintaining a more normal morphology. To further understand the cellular response to hypoxia, we examined the expression of HIF-1α. In normoxic condition, HIF-1α expression was low or negligible. However, in hypoxia-exposed hiPSC-CMs, HIF-1α expression increased significantly, reflecting the cellular adaptation to low oxygen levels. In hypoxia-exposed hiPSC-CMs treated with levosimendan or its metabolite, HIF-1α expression remained unchanged compared to the hypoxia-alone condition (Figure 2).

**FIGURE 3:**
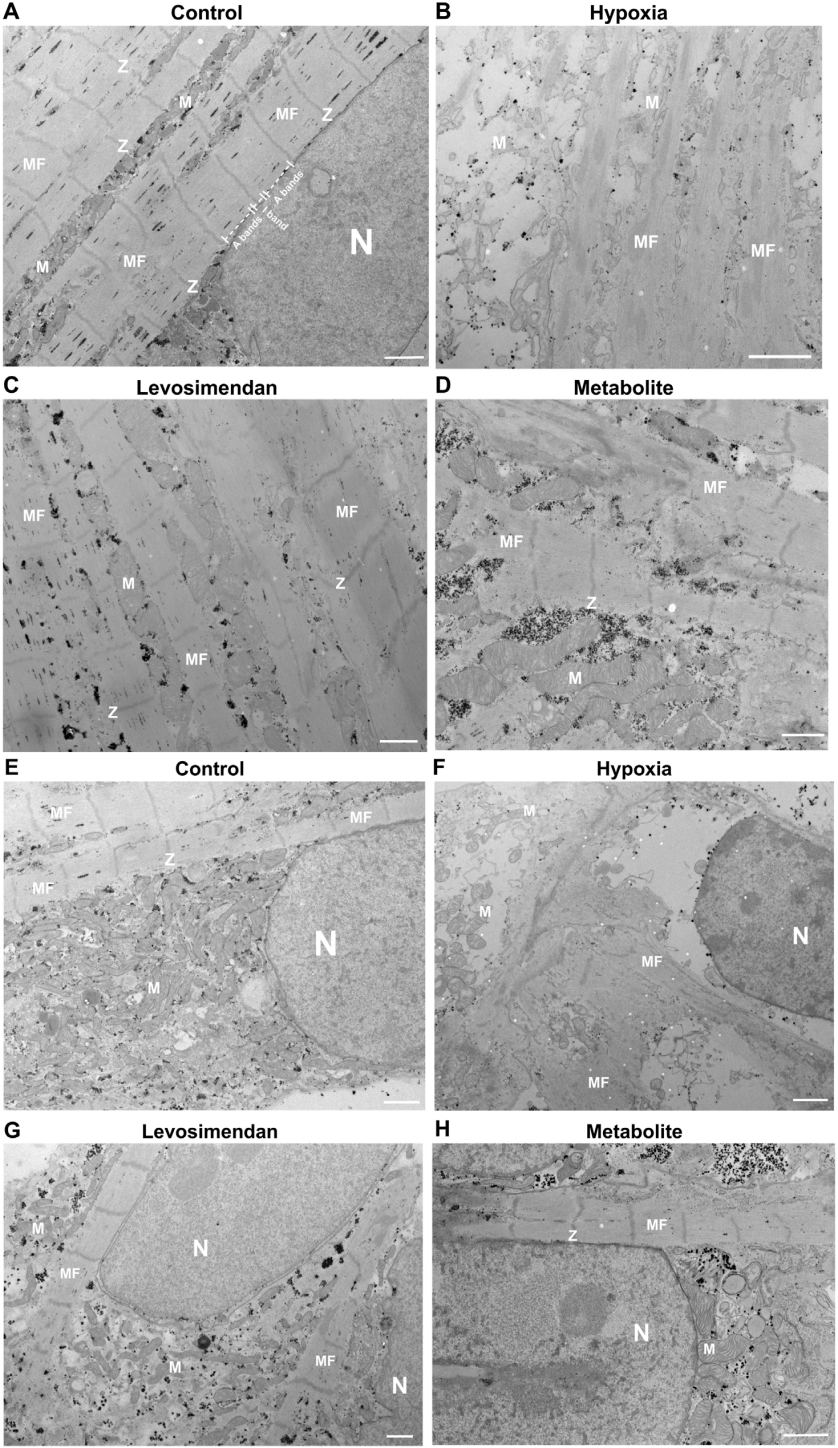
Ultrastructural analysis of hiPSC-CMs. **(A,E)** Representative normoxic hiPSC-CMs showing well-organized sarcomeres, intact Z-discs, and normal mitochondrial and nuclear morphology. **(B,F)** Representative hypoxia-exposed hiPSC-CMs exhibiting sarcomere disorganization, fragmented myofibrils, mitochondrial cristae fragmentation, and nuclear shrinkage. **(C,G)** Representative hypoxia-exposed hiPSC-CMs treated with levosimendan, and **(D,H)** Representative hypoxia-exposed hiPSC-CMs treated with levosimendan metabolite, showing preserved sarcomere organization, intact Z-discs, and maintained mitochondrial and nuclear integrity. Observed features include mitochondria (M), Z-line (Z), myofilaments (MF), and the nucleus (N). The scale bar represents 1 µm. *n* = 3 independent differentiations, with at least 20 random fields analyzed per replicate.

At the ultrastructural level, normoxic hiPSC-CMs exhibited well-maintained integrity, with properly aligned sarcomeres, intact Z-discs, and organized myofibrils (Figure 3A). Mitochondria displayed intact cristae and consistent size, while the nucleus showed normal morphology with evenly distributed chromatin (Figure 3E). In contrast, hypoxia-exposed hiPSC-CMs exhibited significant ultrastructural damage, including misaligned or degraded Z-discs, fragmented myofibrils (Figure 3B), mitochondrial cristae fragmentation, and compromised membrane integrity. Nuclear morphology was also affected, with chromatin condensation and nuclear shrinkage (Figure 3F). We captured an image demonstrating the overall condition of CMs under hypoxic stress, highlighting the massive damage to cellular structures and the dramatic changes induced by hypoxia (Supplementary Figure 4). Notably, hypoxia-exposed hiPSC-CMs treated with levosimendan or its metabolite preserved the ultrastructure of hiPSC-CMs. Sarcomeres remained better organized, with largely intact Z-discs and reduced myofibrillar damage (Figures 3C,D). Mitochondria maintained their size, cristae organization, and membrane integrity, closely resembling those in normoxic conditions. Nuclear morphology was also preserved, with minimal signs of stress, similar to normoxia (Figures 3G,H). In conclusion, hypoxia induces significant structural abnormalities in hiPSC-CMs, including sarcomere disorganization and mitochondrial damage. However, treatment with levosimendan or its metabolite significantly mitigates these effects, resulting in structural features that closely resemble those of normoxic cells. These findings highlight the protective role of levosimendan and its metabolite in preserving CM integrity under hypoxic stress.

### 3.2 Effect of hypoxia on the calcium cycling of hiPSC-CMs and the protective role of levosimendan and its metabolite OR-1896

Abnormalities in Ca^2+^ transients were categorized into the following groups: irregular phase (IP), double peaks (DP), multiple peaks (MP), prolonged rise (PR), and plateau abnormality (PA). Ca^2+^ transients were classified as normal (N) if the hiPSC-CMs exhibited regular beating rhythms with no abnormalities in signal shape or amplitude, and if contraction and relaxation occurred smoothly without any extra Ca^2+^ sparks. Any deviations from this pattern were considered abnormal traces, as previously described (Gaballah et al., 2022). The definitions and the shapes of each abnormality are described in the (Supplementary Figure 5).

**FIGURE 4:**
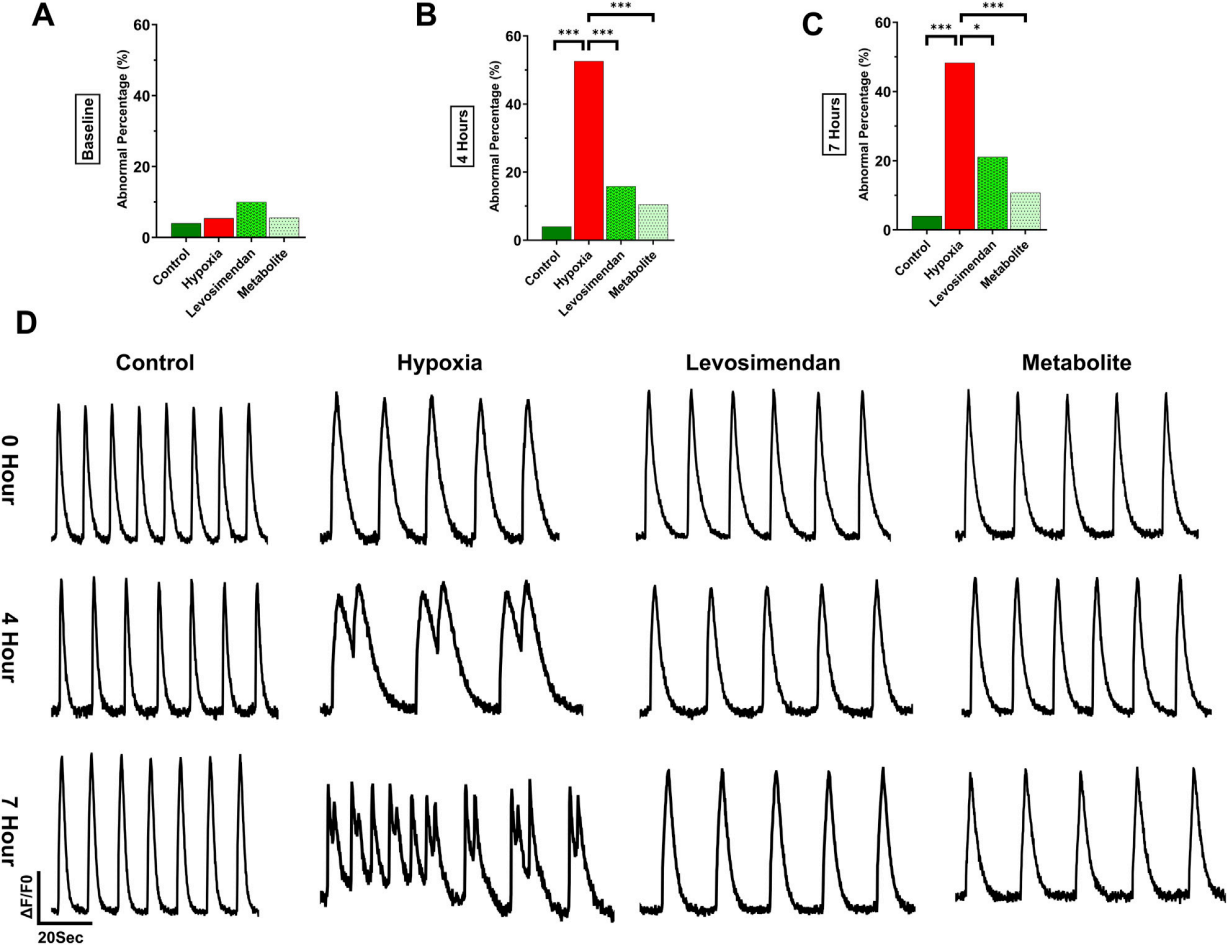
Effect of hypoxia on calcium cycling in hiPSC-CMs and the percentage of calcium transient abnormalities. The figure illustrates the percentage of arrhythmic behavior in normoxic control hiPSC-CMs, hypoxia-exposed hiPSC-CMs, and hypoxia-exposed hiPSC-CMs treated with levosimendan and its metabolite OR-1896. **(A)** Arrhythmic behavior percentage at baseline. **(B)** Arrhythmic behavior percentage at 4 h. **(C)** Arrhythmic behavior percentage at 7 h. **(D)** Representative calcium transient traces in normoxic control hiPSC-CMs, hypoxia-exposed hiPSC-CMs, and hypoxia-exposed hiPSC-CMs treated with levosimendan or its metabolite OR-1896 at baseline, 4h, and 7 h. Data are presented as percentages (%) of the total number of measured abnormalities. Statistical significance: *p < 0.05, **p < 0.01, ***p < 0.001; determined by the non-parametric Kruskal–Wallis test, followed by pairwise comparisons with Dunn’s *post hoc* test and Bonferroni corrections for multiple comparisons. *n* = 5 independent differentiations, with 20–30 calcium transient recordings per well and 3–4 wells per condition.

At baseline, all groups displayed similar behavior, with approximately 5%–10% arrhythmic traces and no statistically significant differences. The normoxic control group maintained consistent behavior throughout the experiment, with an arrhythmic percentage of 4% sustained up to 7 h (Figure 4A). However, the effects of hypoxia on hypoxia-exposed hiPSC-CMs became evident at 4 and 7 h. At 4 h after hypoxia onset, 52.6% of the traces in the hypoxia-exposed hiPSC-CMs group exhibited arrhythmic behavior, which was significantly higher than in the normoxic control, hypoxia-exposed hiPSC-CMs treated with levosimendan (15.8%), or its metabolite OR-1896-treated (11%) groups. Importantly, the slightly higher incidence of arrhythmic behavior in the levosimendan or its metabolite OR-1896 treated groups was not significantly different from the normoxic control (Figure 4B). By 7 h, 48.3% of the traces in the hypoxia-exposed hiPSC-CMs group remained arrhythmic, significantly differing from the normoxic control, hypoxia-exposed hiPSC-CMs treated with levosimendan (21.1%), and metabolite OR-1896 (10.8%) groups. Similar to the 4-h recording, no significant differences were observed between the normoxic control and either of the treatment groups (Figure 4C). Regarding the types of arrhythmic abnormalities recorded by calcium imaging between the different groups at 4 and 7 h, at 4 h, the frequency of double peaks and multiple peaks was notably higher in the hypoxia-exposed hiPSC-CMs group, together accounting for 35.9% of the arrhythmic cases. Notably, hypoxia increased all types of arrhythmic behavior. In the hypoxia-exposed hiPSC-CMs treated with levosimendan or its metabolite, the most common arrhythmic type observed at 4 h was irregular phase, accounting for 7.9% of cases. At 7 h, irregular phase and plateau abnormality were the most prominent arrhythmic behaviors in the hypoxia-exposed hiPSC-CMs group, with multiple peaks levels remaining elevated. In the hypoxia-exposed hiPSC-CMs treated with levosimendan or its metabolite, the most common arrhythmic types observed at 7 h were irregular phase and double peaks (Supplementary Table 1). Statistical analysis confirmed that the reduction in abnormalities in treated with levosimendan or its metabolite groups was significant compared to hypoxia-exposed hiPSC-CMs group.

Under normoxic conditions, hiPSC-CMs exhibited regular calcium transients with normal beating behavior at all imaging time points, including baseline, 4 h, and 7 h. In contrast, induction of hypoxia resulted in a significant increase in calcium transient abnormalities, which became progressively more pronounced over time. By 4 and 7 h of hypoxia exposure, a clear deterioration in calcium handling was observed, characterized by different types of abnormalities, as double peaks, and irregular beating. Notably, hypoxia-exposed hiPSC-CMs treated with levosimendan or its metabolite OR-1896 demonstrated a marked reduction in calcium transient abnormalities. At both 4- and 7-h post-treatment, the majority of cells exhibited normal calcium transients, with regular beating patterns restored (Figure 4D). These results indicate that both levosimendan and its metabolite OR-1896 effectively attenuate hypoxia-induced dysregulation in calcium handling, preserving normal cellular beating properties under hypoxia.

**FIGURE 5:**
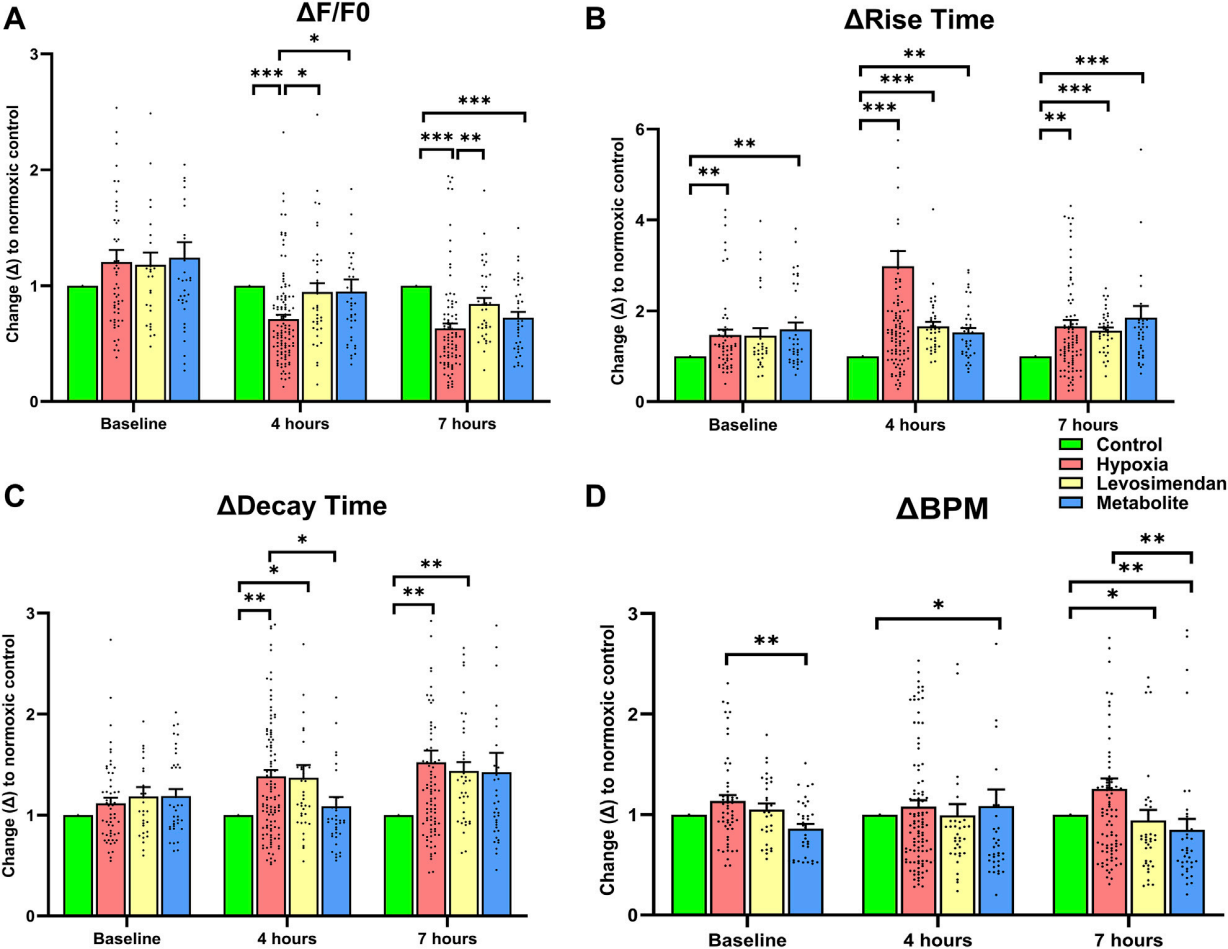
Effects of hypoxia and treatment with levosimendan and its metabolite OR-1896 on Ca^2+^ transient parameters in hiPSC-CMs. Quantitative analysis of normalized calcium transient parameters, including **(A)** Ca^2+^ peak amplitude (relative change in the fluorescence signal from the peak amplitude (F) to the fluorescence baseline (F0); ΔF/F0), **(B)** rise time (time for the Ca^2+^ transient to rise from 10% to 90% of its peak), **(C)** decay time (time for the Ca^2+^ transient to decay from 90% to 10% of its peak), and **(D)** beating rate (beats per minute, BPM). Data are presented as mean ± SEM; statistical significance: *p < 0.05, **p < 0.01, ***p < 0.001; determined by the non-parametric Kruskal–Wallis test, followed by pairwise comparisons with Dunn’s *post hoc* test and Bonferroni corrections for multiple comparisons. *n* = 5 independent differentiations, with 20–30 calcium transient recordings per well and 3–4 wells per condition.

To assess the effects of hypoxia and treatment with either levosimendan or its metabolite on calcium handling, calcium transient parameters were normalized to the normoxic control at each time point. The normalized amplitude of calcium transients showed a significant decrease in the hypoxia-exposed hiPSC-CMs group at both 4 and 7 h compared to the normoxic control. In contrast, hypoxia-exposed hiPSC-CMs treated with levosimendan and its metabolite OR-1896 exhibited a recovery in amplitude, with values approaching near-normoxic levels at 4 and 7 h. Notably, levosimendan sustained calcium transient amplitudes at levels comparable to the normoxic control, differing significantly from both the hypoxia-exposed hiPSC-CMs and levosimendan metabolite -treated groups (Figure 5A). Hypoxia induced a significant increase in the rise time of calcium transients at 4 and 7 h compared to the normoxic control. Treatment with levosimendan and its metabolite partially mitigated this effect, with rise times decreasing toward normoxic levels. However, at both 4 and 7 h, all treatment groups exhibited elevated rise times compared to the normoxic control (Figure 5B). Similarly, decay time was significantly prolonged in the hypoxic group compared to the control group at 4 and 7 h. Treatment with levosimendan and its metabolite reduced decay times, bringing them closer to normoxic levels. At 4 h, the normoxic control and levosimendan metabolite OR-1896-treated groups sustained near-identical decay times, which were significantly shorter than those of the hypoxic control group. By 7 h, the hypoxia-exposed group and the groups treated with levosimendan or its metabolite remained significantly elevated compared to the normoxic control group (Figure 5C). The normalized beating rate (BPM) was slightly higher in the hypoxia-exposed hiPSC-CMs group compared to the normoxic control, as well as the levosimendan- and its metabolite-treated groups, at both 4 and 7 h. Notably, treatment with levosimendan and its metabolite OR-1896 not only reduced the BPM compared to the hypoxic control but also resulted in a significantly lower BPM compared to the normoxic control at both time points (Figure 5D). This suggests that both compounds exert a moderating effect on the beating rate, potentially contributing to their antiarrhythmic properties under hypoxic conditions.

### 3.3 Effect of hypoxia on cardiac biomarkers release in hiPSC-CMs and the modulatory effects of levosimendan and its metabolite OR-1896

**FIGURE 6:**
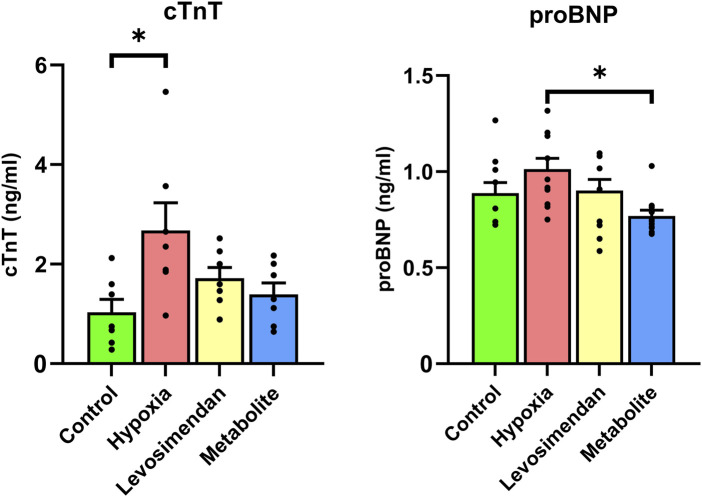
Effects of hypoxia and treatment with levosimendan and its metabolite OR-1896 on cardiac biomarkers release in hiPSC-CMs. ELISA analysis of cardiac troponin T and pro-B-type natriuretic peptide levels in hiPSC-CMs under normoxia, hypoxia, and hypoxia with treatment by levosimendan or its metabolite. Hypoxia-exposed hiPSC-CMs showed an increase in cTnT and proBNP release compared to the normoxic control, which decreased with treatment by levosimendan or its metabolite OR-1896. Data are presented as mean ± SEM; Statistical significance: *p < 0.05, **p < 0.01, ***p < 0.001; determined by one-way ANOVA followed by Tukey’s *post hoc* test. *n* = 3 independent differentiations, with 3 technical replicates per condition.

To evaluate the impact of hypoxia on CM injury and the protective effects of levosimendan and its metabolite, we conducted an ELISA assay to measure the release of cardiac troponin T and pro-B-type natriuretic peptide in hiPSC-CMs, comparing the normoxia group to hypoxia-exposed hiPSC-CMs and hypoxia-exposed hiPSC-CMs treated with levosimendan or its metabolite. The results revealed that hypoxia-exposed cells exhibited a significant increase in the release of cTnT and a non-significant increase in proBNP compared to the normoxic controls. However, treatment with levosimendan or its metabolite markedly reduced the levels of these biomarkers, bringing them closer to those observed in the normoxic control group (Figure 6).

### 3.4 Effect of hypoxia on hypoxia signaling-associated genes in hiPSC-CMs and the modulatory effects of levosimendan and its metabolite OR-1896

**FIGURE 7:**
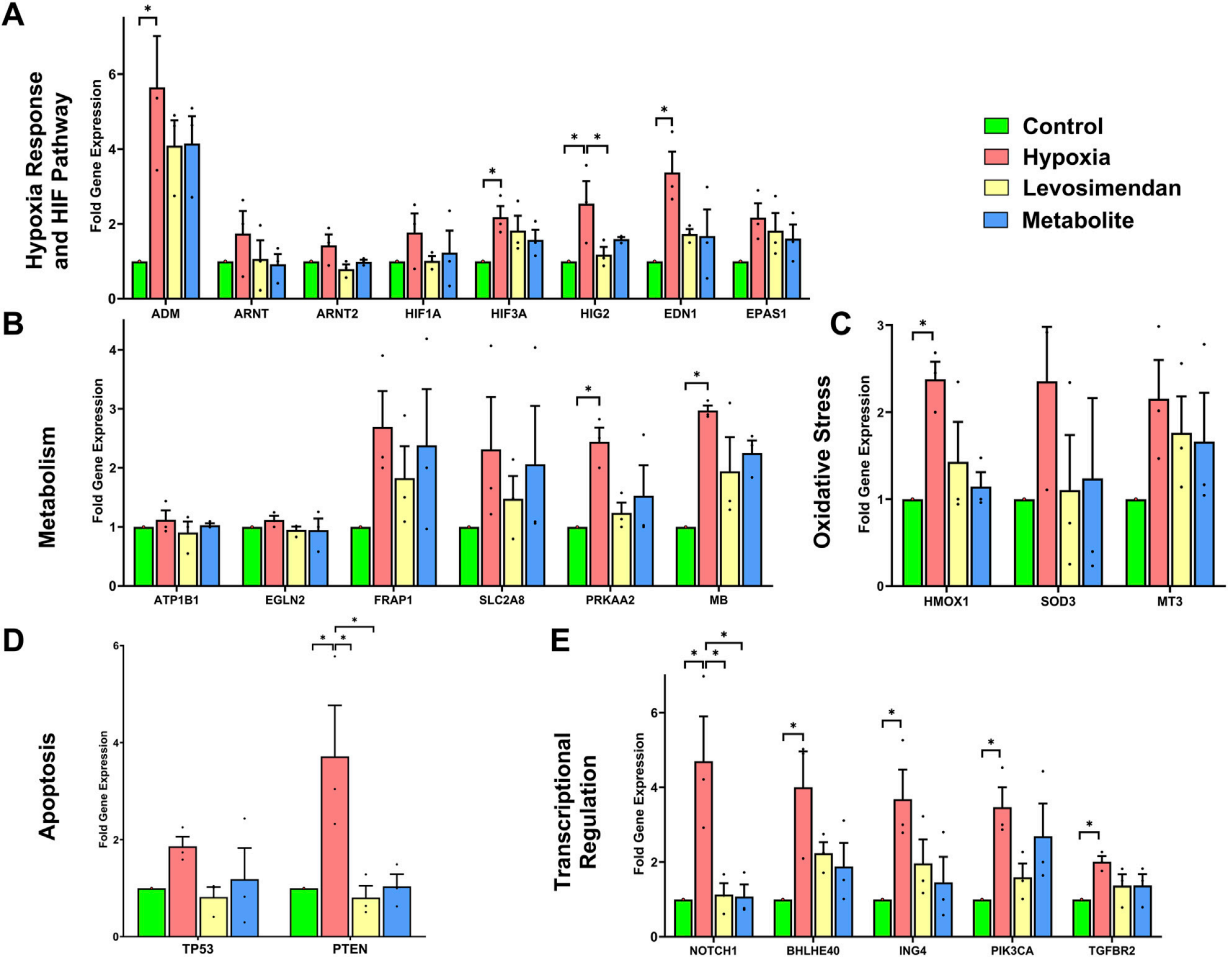
Effects of hypoxia and treatment with levosimendan and its metabolite OR-1896 on gene expression in hiPSC-CMs. **(A)** Hypoxia response and HIF pathway genes. **(B)** Metabolic adaptation genes. **(C)** Oxidative stress response genes. **(D)** Apoptosis-related genes. **(E)** Transcriptional regulation and signaling genes. Hypoxia induced significant upregulation of genes in each category compared to the normoxic control, while treatment with levosimendan or its metabolite OR-1896 resulted in reductions in gene expression. Data are presented as mean ± SEM; Statistical significance: *p < 0.05, **p < 0.01, ***p < 0.001; determined by one-way ANOVA followed by Tukey’s *post hoc* test. *n* = 3 independent differentiations, with 3 technical replicates per condition.

Gene expression analysis was performed using the Human Hypoxia Array Kit to quantify the expression of genes involved in hypoxia response, metabolism, oxidative stress, apoptosis, and transcriptional regulation. Compared to the normoxic control, hypoxia induced significant upregulation of genes associated with the hypoxia response and HIF pathway, including Adrenomedullin (ADM), Hypoxia-Inducible Factor 3 Alpha (HIF3A), Hypoxia-Inducible Protein 2 (HIG2), and Endothelin 1 (EDN1). Additionally, Hypoxia-Inducible Factor 1 Alpha (HIF1A) and Endothelial PAS Domain Protein 1 (EPAS1) levels were elevated under hypoxic conditions. In contrast, Aryl Hydrocarbon Receptor Nuclear Translocator (ARNT) and Aryl Hydrocarbon Receptor Nuclear Translocator 2 (ARNT2) expression showed minimal changes across the groups. Treatment with levosimendan and its metabolite resulted in reductions in the expression of these hypoxia-responsive genes compared to the hypoxia group (Figure 7A). Hypoxia also led to significant upregulation of metabolic adaptation genes, such as Protein Kinase AMP-Activated Catalytic Subunit Alpha 2 (PRKAA2) and Myoglobin (MB), while Mechanistic Target of Rapamycin (FRAP1) and Solute Carrier Family 2 Member 8 (SLC2A8) exhibited moderate increases in expression. In contrast, ATPase Na+/K + Transporting Subunit Beta 1 (ATP1B1) and Egl-9 Family Hypoxia-Inducible Factor 2 (EGLN2) expression remained unchanged across the groups. Treatment with levosimendan and its metabolite resulted in moderate reductions in the expression of these metabolic genes compared to the hypoxia group (Figure 7B). Hypoxia induced clear upregulation of oxidative stress response genes, including Heme Oxygenase 1 (HMOX1), Superoxide Dismutase 3 (SOD3), and Metallothionein 3 (MT3). Treatment with levosimendan and its metabolite resulted in reductions in the expression of these genes compared to the hypoxia group (Figure 7C).

The apoptosis-related genes Phosphatase and Tensin Homolog (PTEN) and Tumor Protein P53 (TP53) exhibited clear increases in expression under hypoxia. Treatment with levosimendan and its metabolite resulted in reductions in the expression of these genes compared to the hypoxia group (Figure 7D). Finally, hypoxia induced significant upregulation of transcriptional regulation and signaling genes, including Basic Helix-Loop-Helix Family Member E40 (BHLHE40), Notch Receptor 1 (NOTCH1), Basic Helix-Loop-Helix Family Member E40 (BHLHE40), Inhibitor of Growth Family Member 4 (ING4), Phosphatidylinositol-4,5-Bisphosphate 3-Kinase Catalytic Subunit Alpha (PIK3CA), and Transforming Growth Factor Beta Receptor 2 (TGFBR2). Treatment with levosimendan and its metabolite resulted in reductions in the expression of these genes compared to the hypoxia group (Figure 7E).

## 4 Discussion

In this study, we investigated the effects of acute hypoxia on hiPSC-CMs and evaluated the cardioprotective and antiarrhythmic properties of levosimendan and its metabolite OR-1896. Our findings reveal that hypoxia induces significant cellular damage, starting with structural disruption. The disruption of sarcomeres and cytoskeletal proteins, such as α-actinin, troponin, and myosin, leading to cell injury and biomarker release, further exacerbates cellular dysfunction by impairing contractility and calcium handling and dysregulated gene expression. Notably, treatment with both levosimendan and its metabolite significantly reduce hypoxia-induced arrhythmogenesis, as evidenced by improved calcium handling, reduced incidence of abnormal Ca^2+^ transients, and mitigated hypoxia-induced alterations in gene expression. Additionally, treatment with levosimendan and its metabolite OR-1896 preserved cell morphology and led to a marked reduction in the release of cardiac biomarkers, such as cTnT and proBNP, indicating protection against hypoxia-induced cellular injury. These results align with our previous studies highlighting levosimendan’s antiarrhythmic and cardioprotective properties (Gaballah et al., 2022), while also providing novel insights into the therapeutic potential of its metabolite, OR-1896.

In the current study, hypoxia induced significant structural and ultrastructural change in hiPSC-CMs, including sarcomere disorganization, mitochondrial damage, and nuclear shrinkage. These changes reflect the severe cellular stress induced by oxygen deprivation, which disrupts the cytoskeletal framework and compromises organelle function. The observed mitochondrial damage, in particular, is a critical contributor to hypoxia-induced injury, as it impairs energy production, increases reactive oxygen species (ROS) generation, and triggers apoptotic pathways (Waypa et al., 2006), (Wu et al., 2018). These findings are consistent with previous studies demonstrating that oxygen deprivation disrupts CM architecture, leading to impaired contractile function (Gaballah et al., 2022).

The structural damage induced by hypoxia was accompanied by a significant increase in the release of cardiac biomarkers, such as cardiac troponin T and pro-B-type natriuretic peptide. These biomarkers are indicative of membrane damage and cellular injury, reflecting the breakdown of sarcolemmal integrity and the activation of stress pathways (Xie et al., 2024), (Casals et al., 2009). The release of cTnT, in particular, is a hallmark of CM injury and is widely used as a diagnostic marker for myocardial infarction (Osredkar et al., 2024), (Wu et al., 2021). The increase in proBNP levels further underscores the severity of hypoxia-induced stress, as this biomarker is released in response to increased wall stress and myocardial stretch (Goetze et al., 2003), (Hall, 2005). Together, the elevation of these biomarkers highlights the profound impact of hypoxia on CM viability and function.

In addition, hypoxia triggered significant changes in gene expression, upregulating pathways associated with the hypoxia response (e.g., HIF-1α, HIF3A, HIG2), oxidative stress (e.g., HMOX1, SOD3, MT3), and apoptosis (e.g., PTEN, TP53). The upregulation of hypoxia response genes reflects the cellular response and adaptation to low oxygen levels (Weidemann and Johnson, 2008), (Ziello et al., 2007), while the increase in oxidative stress markers indicates the accumulation of ROS and the activation of antioxidant defense mechanisms (Kimura et al., 1985), (Giordano, 2005). The upregulation of apoptotic genes further highlights the progression from cellular stress to programmed cell death (Giordano, 2005). These changes in gene expression represent a coordinated but ultimately maladaptive response to hypoxia, aimed at promoting survival under adverse conditions but resulting in further cellular damage.

One of the most critical consequences of hypoxia is the dysregulation of calcium handling, which plays a central role in arrhythmogenesis. The disruption of sarcomeres and cytoskeletal proteins, such as α-actinin, troponin, and myosin, further exacerbates cellular dysfunction by impairing contractility and calcium handling. Hypoxia-induced calcium overload disrupts the balance between calcium influx and efflux, leading to spontaneous depolarizations and abnormal calcium transients (Barry, 2006). In our study, hypoxia-exposed hiPSC-CMs exhibited a high incidence of arrhythmic events, including double peaks, multiple peaks, and irregular phases, reflecting the destabilization of calcium homeostasis. Calcium dysregulation is closely linked to mitochondrial dysfunction, as impaired ATP production reduces the activity of calcium pumps and exchangers, exacerbating calcium overload (Barry, 2006). This creates a feedback loop, where calcium overload further damages mitochondria, leading to increased ROS production and cellular injury (Adzigbl et al., 2022). These findings are in line with previous studies highlighting hypoxia-induced calcium dysregulation as a major contributor to cardiac dysfunction (Gorski et al., 2015), (Arnaud et al., 2023).

Treatment with levosimendan and its metabolite OR-1896 significantly reduced the effects of hypoxia, preserving cellular structure, reducing biomarker release, and stabilizing calcium transients, closely resembling normoxic conditions. Levosimendan and its metabolite OR-1896 maintained sarcomere alignment, mitochondrial integrity, and nuclear morphology, preventing the structural damage induced by hypoxia. The preservation of mitochondrial integrity is particularly noteworthy, as mitochondria play a central role in energy production and calcium homeostasis (Zhang et al., 2022). By maintaining mitochondrial structure, levosimendan and its metabolite OR-1896 likely prevent the collapse of the mitochondrial membrane potential, reduce reactive oxygen species (ROS) production, and inhibit the opening of the mitochondrial permeability transition pore (mPTP), all of which are key contributors to hypoxia-induced cell death. This mode of action aligns with the known ability of levosimendan to enhance mitochondrial efficiency and reduce oxidative stress in CMs (Pathak et al., 2013), (Susilo et al., 2024).

The reduction in cTnT and proBNP release from hiPSC-CMs suggests that levosimendan and its metabolite OR-1896 stabilize the sarcolemma and inhibit the activation of stress pathways. This reduction in biomarker release underscores the cardioprotective effects of both compounds, suggesting the ability of levosimendan and its metabolite OR-1896 to prevent the disruption of membrane integrity under hypoxic conditions. This protective effect may be mediated by their ability to enhance mitochondrial function, reduce ROS production, and inhibit the opening of the mitochondrial permeability transition pore (Caimmi et al., 2011). Additionally, these compounds may inhibit the activation of proteases and phospholipases that contribute to cellular injury. This mode of action is consistent with the known role of levosimendan in enhancing CM survival during ischemia-reperfusion injury (Pollesello and Papp, 2007). These findings align with previous clinical and experimental studies demonstrating levosimendan’s cardioprotective effects in ischemic conditions (Gaballah et al., 2022), (Kiraz et al., 2015), (Li et al., 2014).

Levosimendan and its metabolite OR-1896 also modulated the expression of hypoxia-responsive and oxidative stress genes, downregulating HIF-1α, HMOX1, SOD3, and other markers of cellular stress. This suggests that levosimendan and its metabolite OR-1896 interfere with hypoxia signaling pathways, thereby attenuating cellular stress responses. HIF-1α is a key regulator of cellular adaptation to low oxygen levels, and its downregulation may reflect improved oxygen utilization and reduced cellular stress (Weidemann and Johnson, 2008). Similarly, the reduction in oxidative stress markers, such as HMOX1 and SOD3, indicates that both compounds enhance the cellular antioxidant defense system, thereby reducing ROS accumulation and preventing oxidative damage to proteins, lipids, and DNA (Pathak et al., 2013) and further supports their role in CM survival under hypoxic conditions. The downregulation of apoptotic genes, such as PTEN and TP53, further highlights the ability of levosimendan and its metabolite OR-1896 to inhibit programmed cell death and promote CM survival. These effects are consistent with the known role of levosimendan in enhancing mitochondrial efficiency and reducing oxidative stress in CMs (Pollesello and Papp, 2007), (Antila et al., 2007), (Grossini et al., 2020). The observed gene expression changes provide insights into the molecular mechanisms underlying the protective effects of levosimendan and its metabolite OR-1896, further supporting their potential as therapeutic agents.

One of the most striking findings of this study is the ability of levosimendan and its metabolite OR-1896 to reduce the incidence of abnormal Ca^2+^ transients and restore normal calcium handling under hypoxic conditions. Notably, in addition to their cardioprotective effects, treatment with levosimendan and its metabolite significantly reduced arrhythmic events and preserved normal calcium transient parameters. This protective effect is likely mediated through levosimendan’s ability to enhance calcium sensitivity of troponin C and its impact on mitochondrial ATP production, which supports cellular energy demands under stress conditions also which could be attributed to their ability to stabilize calcium handling and improve myocardial contraction (Li et al., 2014), (Antila et al., 2007). This mechanism likely contributes to the stabilization of calcium transients and the reduction in arrhythmic events observed in this study. These findings are consistent with previous studies demonstrating the antiarrhythmic effects of levosimendan in ischemic conditions (Gaballah et al., 2022), (Heringlake et al., 2021), (Kowalczyk et al., 2010). The ability of both compounds to reduce abnormal calcium transients, such as double peaks and irregular phases, highlights their potential as antiarrhythmic agents.

While the cardioprotective effects of levosimendan have been well-documented, this study is the first to demonstrate that its metabolite, OR-1896, exhibits similar or even enhanced protective properties. Levosimendan metabolite OR-1896 not only maintained cellular structure and function but also regulated gene expression and reduced biomarker release to a level similar to that of levosimendan. This is particularly important given levosimendan meatbolite OR-1896s extended half-life (70–80 h for the metabolite OR-1896s compared to 1 h for the parent drug, levosimendan), which may contribute to maintain prolonged therapeutic effects in clinical applications (Antila et al., 2007). In addition, Levosimendan and its metabolite OR-1896 exert cardioprotective effects through modulation of mitochondrial ATP-sensitive potassium channels and preservation of mitochondrial membrane potential (Papp et al., 2012). The observed effects of levosimendan metabolite OR-1896 on calcium handling, oxidative stress, and gene expression suggest that it may act through multiple pathways, including the stabilization of calcium homeostasis, enhancement of mitochondrial function, and modulation of hypoxia-responsive signaling. This highlights the potential of levosimendan metabolite OR-1896 as a novel therapeutic agent for managing ischemia-induced arrhythmias.

Finally, our findings suggest that levosimendan and its metabolite OR-1896 may provide therapeutic benefits under hypoxic conditions by preserving CM structure and function, highlighting their potential use in managing hypoxia-related cardiac disorders. While levosimendan is already clinically used for acute heart failure, our study emphasizes the need for further research into its potential cardioprotective role during myocardial ischemia. Future studies should focus on elucidating the precise molecular mechanisms underlying these protective effects and should explore the long-term effects and potential clinical applications of these compounds in ischemic heart conditions and evaluating their translational potential in in vivo models and clinical settings.

## 5 Conclusion

In conclusion, our study demonstrates that acute hypoxia induces a cascade of cellular damage in hiPSC-CMs, beginning with structural disruption, progressing to cell injury and biomarker release, and ultimately leading to dysregulated gene expression and impaired calcium handling. Treatment with levosimendan and its metabolite OR-1896 effectively reduced these effects, preserving cellular integrity and function. Since levosimendan is already used in heart failure management and is rapidly metabolized into levosimendan metabolite OR-1896, which prolongs its therapeutic action, our findings highlight levosimendan metabolite OR-1896s potential as a cardioprotective agent against ischemia-induced arrhythmias and cellular injury. Further studies are warranted to validate these findings in more complex models and assess the clinical relevance of levosimendan metabolite OR-1896 in ischemic heart disease.

## 6 Study limitations and future perspectives

Despite these promising results, our study has certain limitations. First, while the hiPSC-CM model is highly valuable for studying human cardiac physiology, it does not fully replicate the maturity and complexity of adult CMs. Moreover, we did not distinguish between CM subtypes (atrial, nodal, or ventricular), and the presence of other cell types cannot be ruled out. Second, the experiments were conducted in 2D conditions rather than 3D, which more closely resemble the native cellular environment of human CMs. Future studies should consider employing more advanced models, such as engineered heart tissues or organ-on-chip technology, to further validate these findings. Third, the long-term effects of Levosimendan and its metabolite were not evaluated, underscoring the need for further studies in chronic hypoxia models. Fourth, a more in-depth investigation into the molecular mechanisms underlying the antiarrhythmic effects of OR-1896, particularly its interactions with calcium-handling proteins and ion channels, would enhance our understanding of its therapeutic potential. Fifth, incorporation of a reperfusion phase could substantially improve the clinical relevance of the model, as ischemia-reperfusion injury more closely mimics myocardial infarction and allows assessment of the full cardioprotective potential of the compounds. Sixth, the lack of electrical pacing in our calcium imaging experiments led to variability in spontaneous beat rates across samples; future studies could include pacing protocols to standardize beat frequency and improve the consistency of functional measurements. Seventh, the measurement of cell and nuclear areas, and presentation of these data in graphical form, was not performed; this would have strengthened the structural analysis. Eighth, we restricted our analyses to the functional and structural changes observed after 24 h of hypoxia, without assessing CM viability or necrosis. Although this exposure window has been widely reported to induce robust electrophysiological and metabolic alterations while maintaining cell viability, we did not extend hypoxia beyond this period, nor did we quantify cell death under either hypoxia alone or in the presence of Levosimendan/OR-1896. In addition, protein-level readouts and longer-term hypoxia/reperfusion experiments were not incorporated, which would provide a more comprehensive view of the molecular pathways and chronic adaptations induced by these compounds. Future work should therefore systematically examine prolonged hypoxia and reperfusion models, together with protein-level analyses and viability assessments. Ninth, electrophysiological properties were not comprehensively assessed using patch-clamp or voltage-sensitive dye assays, which would provide more detailed functional characterization. Tenth, metabolic fluxes such as lactate, pyruvate, and glucose production, as well as Seahorse-based bioenergetic measurements, were not evaluated; these would offer valuable insights into the metabolic and bioenergetic effects of Levosimendan and OR-1896. Finally, Levosimendan and OR-1896 were administered to CMs prior to hypoxia induction, thereby modeling a prophylactic rather than a therapeutic intervention. While this approach allowed us to assess the preventive, cardioprotective effects of these compounds, it does not reflect the typical clinical scenario where ischemia/hypoxia occurs unexpectedly and treatment is usually initiated afterwards. Consequently, the translational interpretation of our findings is limited, as the ability of Levosimendan and OR-1896 to reverse or attenuate established hypoxia-induced damage remains unaddressed. Future studies should specifically investigate the post-hypoxia administration of these compounds to better evaluate their therapeutic potential in clinically relevant settings.

## Data Availability

The original contributions presented in the study are included in the article/Supplementary Material, further inquiries can be directed to the corresponding author.
