# Supplementary material for: A hypoxia-on-a-chip platform for modeling ischemic arrhythmogenesis and evaluating the effects of levosimendan and OR-1896 on ischemic human iPSC-derived cardiomyocytes
Source: Front Bioeng Biotechnol. 2025 Sep 29;13:1671013. doi: 10.3389/fbioe.2025.1671013 (PMC12515882; doi:10.3389/fbioe.2025.1671013)
Supplement: Supplementary file 1 [file DataSheet1.pdf]

# A Hypoxia-on-a-Chip Platform for Modeling Ischemic Arrhythmogenesis and Evaluating the Effects of Levosimendan and OR-1896 on Ischemic Human iPSC-Derived Cardiomyocytes

**Mahmoud Gaballah[1,2*], Kaisla Walls[3‡], Fatma Zakzook[1,4‡], Joose Kreutzer[5]**
**Jouko Levijoki[6], Katriina Aalto-Setälä[1,7]**

[1]Heart Group, Faculty of Medicine and Health Technology, Tampere University, Tampere, Finland

[2]Department of Forensic Medicine and Toxicology, Faculty of Veterinary Medicine, University of Sadat City, Menoufia, Egypt

[3]Computational Biophysics and Imaging Group, Faculty of Medicine and Health Technology, Tampere University, Tampere, Finland

[4]Department of Forensic Medicine and Toxicology, Faculty of Veterinary Medicine, Kafrelsheikh University, Kafrelsheikh, Egypt

[5]BioGenium Microsystems Ltd, Tampere, Finland

[6]Orion Corporation Orion Pharma, Espoo, Finland

[7]Heart Hospital, Tampere University Hospital, Tampere, Finland

*Corresponding author: Mahmoud Gaballah (*mahmoud.gaballah@tuni.fi*)

‡ These authors contributed equally as second authors

# Supplementary Material

## - hiPSCs Differentiation

Cardiomyocyte differentiation was performed using the embryoid body (EB) method. On day 0, hiPSCs were detached using Versene (Gibco) and suspended in KSR medium containing 5 µM Blebbistatin (Sigma). The cells were then transferred to ultra-low attachment 6-well plates to facilitate EB formation. On day 1, the medium was replaced with differentiation medium, consisting of RPMI 1640 + GlutaMAX supplemented medium (Gibco) containing 1% B-27 Minus Insulin (50X, Gibco) and 0.5% penicillin/streptomycin, supplemented with 5 µg/ml ascorbic acid (Sigma), 10 ng/ml bone morphogenic protein 4 (BMP4, R&D Systems), and 25 ng/ml Activin A (Peprotech). On day 3, the medium was replaced with fresh differentiation medium supplemented with 5 µg/ml ascorbic acid. On day 4, the medium was exchanged with differentiation medium containing 2.5 µM IWP-4 (Tocris) and 5 µg/ml ascorbic acid. On day 7, half of the medium was replaced with differentiation medium supplemented with 5 µg/ml ascorbic acid. From day 9 onward, half of the medium was exchanged three times per week. Starting on day 11, the differentiation medium was supplemented with B-27 Supplement (50X, serum-free, Gibco). The differentiation protocol is illustrated in Figure 1.

## - Optimization of Levosimendan concentration

The working concentration of 2 µM Levosimendan used in this study was selected based on both prior literature and our preliminary titration experiments on hiPSC-CMs. Previous reports have demonstrated that 2 µM provides robust cardioprotective effects in hiPSC-CMs and adult cardiomyocytes without inducing cytotoxicity. In our preliminary optimization experiments, this concentration was well tolerated and reproducibly enhanced contractility, calcium handling, and mitochondrial performance under ischemic stress. Based on these findings, we selected 2 µM as the optimal balance between efficacy and safety for all experiments in this study.

## - Immunostaining

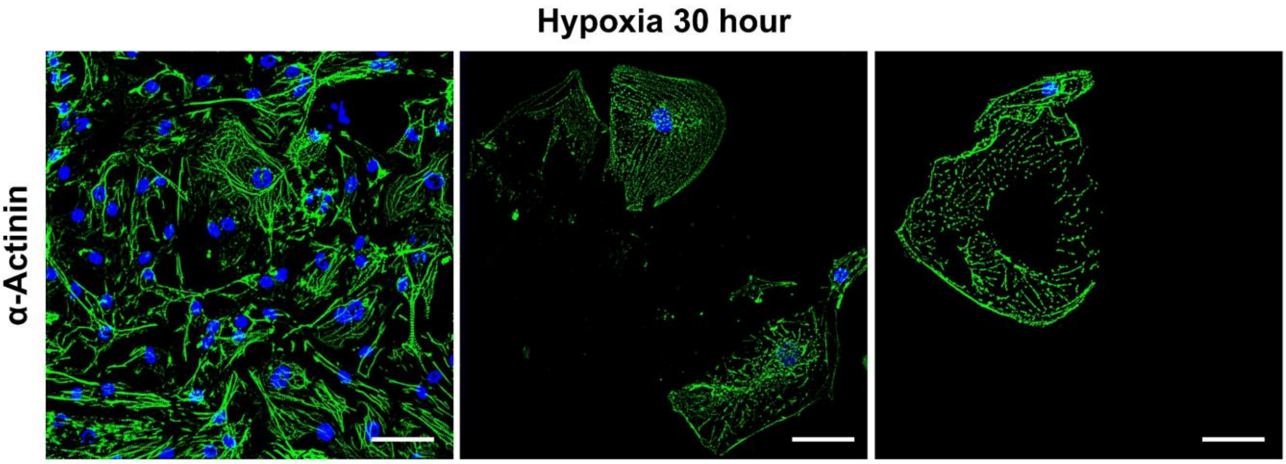


**Figure (S1): Immunostaining of α-actinin in hiPSC-CMs under prolonged hypoxia.** Representative image of hiPSC-CMs after 30 hours of hypoxia. Prolonged exposure caused extensive cell death, with marked loss of sarcomeric structures (α-actinin, green), cytoskeletal disorganization, nuclear loss (DAPI, blue), and, in some regions, complete detachment of cardiomyocytes. These findings highlight the severe damage associated with longer hypoxia durations and support the choice of 24-hour exposure as the experimental window for functional analyses. Scale bar: 20 µm.

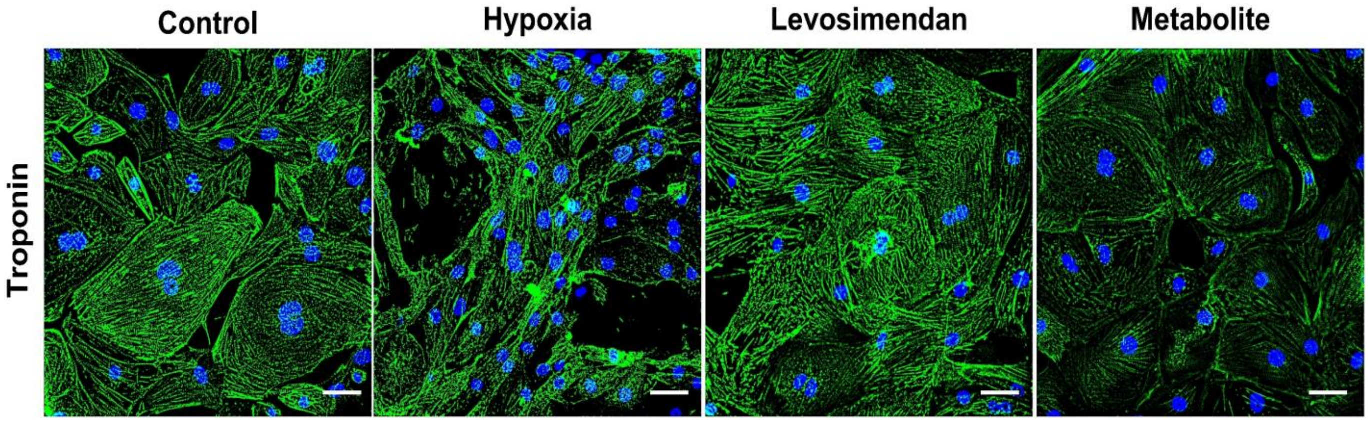


**Figure (S2): Immunostaining of troponin T in hiPSC-CMs.** Representative image of troponin T staining in normoxic hiPSC-CMs, hypoxia-exposed hiPSC-CMs, and hypoxia-exposed hiPSC-CMs treated with levosimendan or its metabolite. The scale bar represents 20 µm.

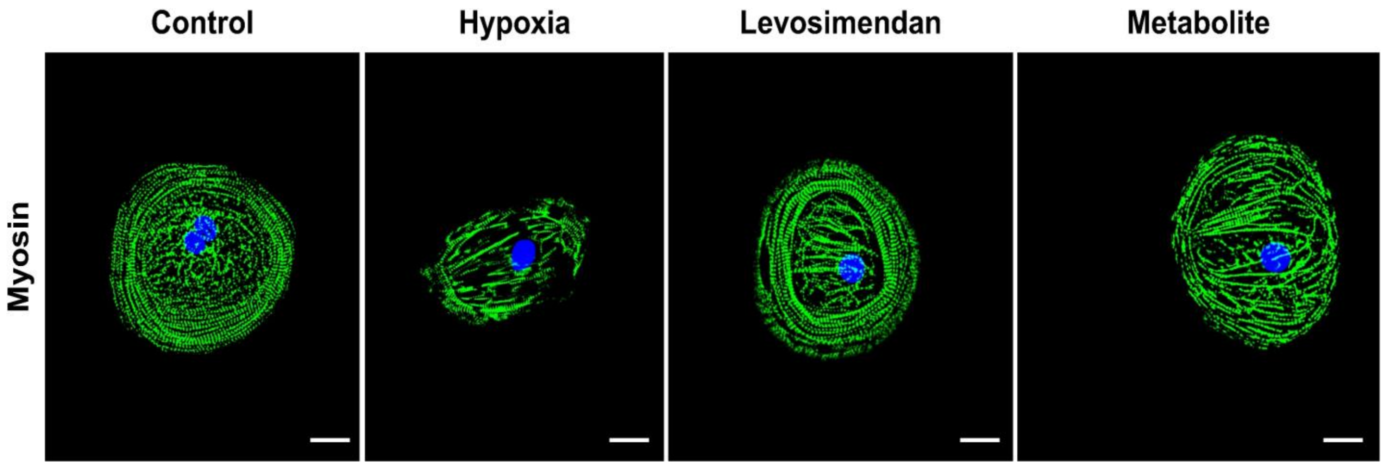


**Figure (S3): Immunostaining of myosin-binding protein C in hiPSC-CMs.** Representative images of myosin-binding protein C staining in normoxic hiPSC-CMs, hypoxia-exposed hiPSC-CMs, and hypoxia-exposed hiPSC-CMs treated with levosimendan or its metabolite. The scale bar represents 20 µm.

- **<u>Ultrastructural analysis of hiPSC-CMs</u>**

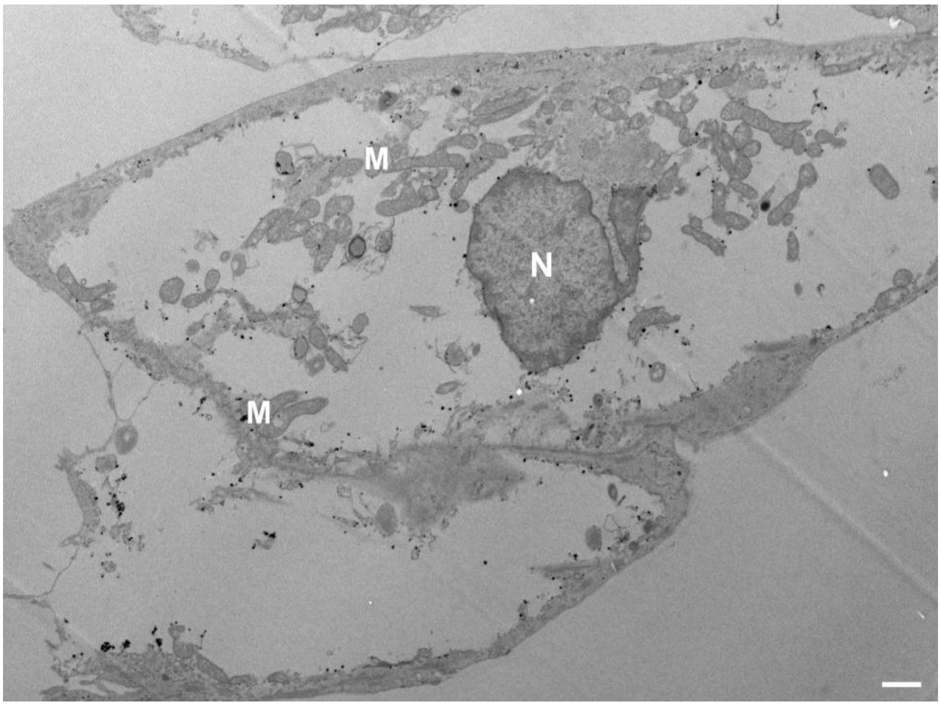


**Figure (S4): Ultrastructural analysis of hiPSC-CMs under hypoxic stress.** Representative electron microscopy image showing the overall condition of a cardiomyocyte under hypoxic stress, highlighting extensive structural damage, including sarcomere disorganization, mitochondrial fragmentation, and nuclear shrinkage. The scale bar represents 1 µm.

## - $Ca^{2+}$ Imaging

In the $Ca^{2+}$ imaging experiments, hiPSC-CMs exhibited abnormal $Ca^{2+}$ transients under hypoxic conditions, which were categorized into the following groups: irregular phase, double peaks, multiple peaks, prolonged rise, and plateau abnormality. $Ca^{2+}$ traces were classified as normal (N) if the hiPSC-CMs displayed regular rhythms, with contraction and relaxation phases occurring smoothly without delays or additional movements. Any deviations from this pattern were considered abnormal traces.

Abnormal $Ca^{2+}$ transients were categorized as follows:

- **Irregular phase (IP):** Unequal intervals between $Ca^{2+}$ transients.
- **Double peaks (DP):** Two successive peaks in the same transient without decaying to the baseline.
- **Multiple peaks (MP):** Three or more successive peaks in the same transient without decaying to the baseline.
- **Plateau abnormality (PA):** Prolonged decay time of the $Ca^{2+}$ transient, characterized by a notch in the decaying phase.
- **Prolonged rise (PR):** Prolonged rise time of the $Ca^{2+}$ transient, characterized by a notch in the rising phase.

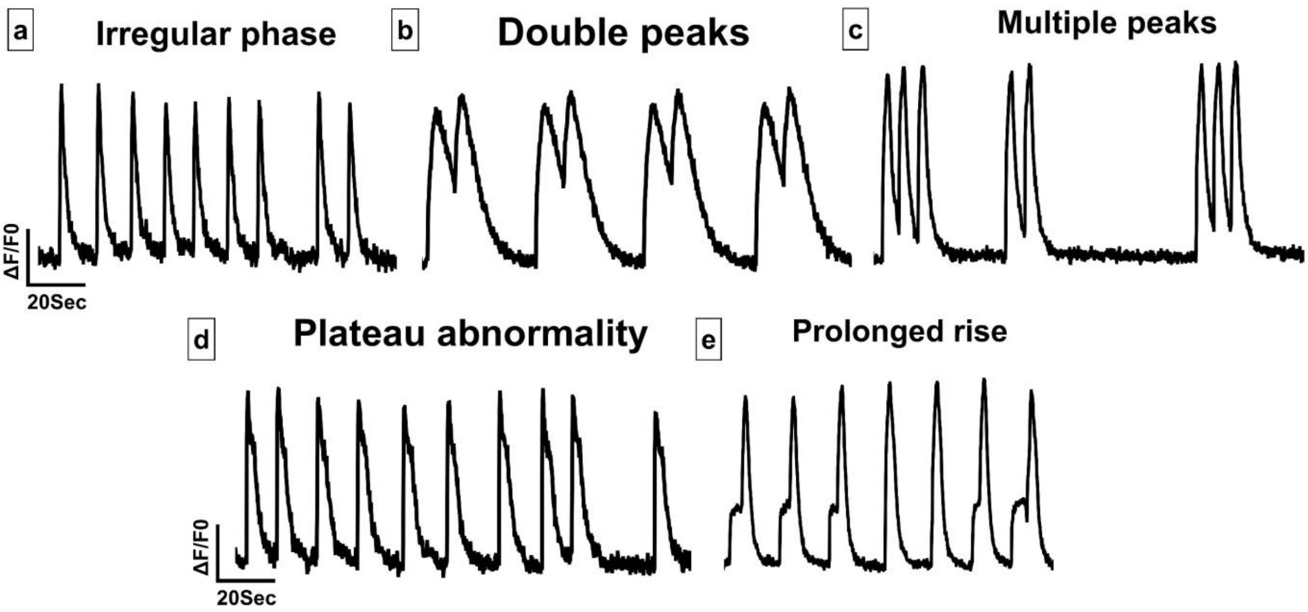


**Figure (S5): Types of $Ca^{2+}$ transient abnormalities in hiPSC-CMs.** Abnormal $Ca^{2+}$ traces showing (a) irregular phase, (b) double peaks, (c) multiple peaks, (d) plateau abnormality, and (e) prolonged rise.

**Table (S1): Calcium transient abnormality types in hiPSC-CMs.** Percentages of each type of abnormality (irregular phase, double peaks, multiple peaks, prolonged rise, and plateau abnormality) in normoxic control, hypoxia-exposed hiPSC-CMs, and hypoxia-exposed hiPSC-CMs treated with levosimendan and its metabolite at baseline, 4 hours, and 7 hours.

| Time | Group | Normal Percentage | Classification and Percentages of Calcium Transient Abnormalities | | | | | |
|---|---|---|---|---|---|---|---|---|
| | | | Irregular phase | Double peaks | Multiple peaks | Plateau abnormality | Prolonged rise | Overall Abnormality |
| Baseline (0 hour) | Normoxic control | 96% | - | - | - | - | 4% | 4% |
| | Hypoxia | 94.6% | 1.8% | - | - | - | 3.6% | 5.4% |
| | Levosimendan | 90% | 3.3% | - | - | 3.3% | 3.3% | 10% |
| | Metabolite OR-1896 | 94.4% | 2.8% | 2.8% | - | - | - | 5.6% |
| 4 hour | Normoxic control | 96% | 2% | 2% | - | - | - | 4% |
| | Hypoxia | 47.4% | 9.6% | 21.9% | 14% | 3.5% | 3.5% | 52.6% |
| | Levosimendan | 84.2% | 7.9% | 2.6% | 2.6% | 2.6% | - | 15.8% |
| | Metabolite OR-1896 | 89.5% | 7.9% | - | 2.6% | - | - | 10.5% |
| 7 hour | Normoxic control | 95.8% | 2.1% | - | - | 2.1% | - | 4.2% |
| | Hypoxia | 51.7% | 16.9% | 4.5% | 9% | 13.5% | 4.5% | 48.3% |
| | Levosimendan | 78.9% | 13.2% | 2.6% | 2.6% | 2.6% | - | 21.1% |
| | Metabolite OR-1896 | 89.2% | 2.7% | 5.4% | 2.7% | - | - | 10.8% |

**Table (S2): List of genes included in the TaqMan® Array Human Hypoxia used for quantitative PCR analysis**

Genes are grouped according to their primary biological functions: Hypoxia response and HIF pathway, Metabolism and Oxidative stress, Apoptosis, and Transcriptional regulation.

| Gene | Name |
|---|---|
| **Housekeeping gene** | |
| **GAPDH** | Glyceraldehyde-3-Phosphate Dehydrogenase gene |
| **Hypoxia response and HIF pathway** | |
| **ADM** | Adrenomedullin gene |
| **ARNT** | Aryl Hydrocarbon Receptor Nuclear Translocator gene (HIF-1β) gene |
| **ARNT2** | Aryl Hydrocarbon Receptor Nuclear Translocator 2 gene (HIF-2β) gene |
| **EDN1** | Endothelin 1 gene |
| **EPAS1** | Endothelial PAS Domain Protein 1 (also known as HIF-2α) gene |
| **HIF1A** | Hypoxia-Inducible Factor 1 Subunit Alpha gene |
| **HIF3A** | Hypoxia Inducible Factor 3 Subunit Alpha gene |
| **HIG2** | Hypoxia Inducible Lipid Droplet-Associated gene |
| **Metabolism** | |
| **ATP1B1** | ATPase $Na^+/K^+$ Transporting Subunit Beta 1 gene |
| **EGLN2** | Egl-9 Family Hypoxia Inducible Factor 2 gene |
| **FRAP1** | FK506 Binding Protein 12-Rapamycin Complex-Associated Protein 1 gene |
| **MB** | Myoglobin gene |
| **PRKAA2** | Protein Kinase AMP-Activated Catalytic Subunit Alpha 2 gene |
| **SLC2A8** | Solute Carrier Family 2 Member 8 gene |
| **Oxidative stress** | |
| **HMOX1** | Heme Oxygenase 1 gene |
| **MT3** | Metallothionein 3 gene |
| **SOD3** | Superoxide Dismutase 3 gene |
| **Apoptosis** | |
| **PTEN** | Phosphatase and Tensin Homolog gene |
| **TP53** | Tumor Protein P53 gene |
| **Transcriptional regulation** | |
| **BHLHE40** | Basic Helix-Loop-Helix Family Member E40 gene |
| **ING4** | Inhibitor of Growth Family Member 4 gene |
| **NOTCH1** | Notch Receptor 1 gene |
| **PIK3CA** | Phosphatidylinositol-4,5-Bisphosphate 3-Kinase Catalytic Subunit Alpha gene |
| **TGFBR2** | Transforming Growth Factor Beta Receptor 2 gene |
